# Supplementary material for: MetaRibo-Seq measures translation in microbiomes
Source: Nat Commun. 2020 Jun 29;11:3268. doi: 10.1038/s41467-020-17081-z (PMC7324362; doi:10.1038/s41467-020-17081-z)
Supplement: Supplementary file 10 — Supplementary Data 7 [file 41467_2020_17081_MOESM10_ESM.zip › File2/Confidence_High_Taxonomy/132205_out.krona.html]

Javascript must be enabled to view this page.

members
magnitude
magnitudeUnassigned
count
unassigned
taxon
rank

132205\_out

310

superkingdom
310
2

310
phylum
976

200643
class
310

310
order
171549

815
family
306


SRS015264\_contig\_number\_684SRS015782\_contig\_number\_16601SRS015960\_contig\_number\_944SRS017433\_contig\_number\_contig-100\_1015.106782SRS020869\_contig\_number\_29997SRS023583\_contig\_number\_558SRS024075\_contig\_number\_10000SRS048870\_contig\_number\_3399SRS050026\_contig\_number\_1203SRS051882\_contig\_number\_1014SRS054059\_contig\_number\_contig-100\_811.46181SRS064557\_contig\_number\_3703SRS064645\_contig\_number\_contig-100\_628.628SRS074586\_contig\_number\_5064SRS077086\_contig\_number\_12892SRS077641\_contig\_number\_20967SRS104084\_contig\_number\_1103SRS1041141\_contig\_number\_1253SRS105153\_contig\_number\_20181SRS142599\_contig\_number\_911SRS142980\_contig\_number\_2199SRS143181\_contig\_number\_13114SRS146812\_contig\_number\_58095SRS146813\_contig\_number\_5279SRS148319\_contig\_number\_15702SRS149244\_contig\_number\_270SRS893292\_contig\_number\_662SRS971275\_contig\_number\_43476
306
28
816
genus


SRS019910\_contig\_number\_12SRS147766\_contig\_number\_239SRS893327\_contig\_number\_7826
47678
3
species

1896973

SRS065397\_contig\_number\_41
1
species


SRS024388\_contig\_number\_110SRS1041132\_contig\_number\_1188
671267
2
species

818

SRS021153\_contig\_number\_422
species
1


SRS063127\_contig\_number\_698
1262750
species
1

1
species
371601

SRS147271\_contig\_number\_81

1263056

SRS014459\_contig\_number\_contig-100\_464.231851SRS015065\_contig\_number\_contig-100\_364.221790SRS143523\_contig\_number\_15429
3
species


SRS064232\_contig\_number\_700SRS1054691\_contig\_number\_5955
2292935
species
2


SRS014736\_contig\_number\_165SRS019267\_contig\_number\_6225SRS050941\_contig\_number\_25SRS055966\_contig\_number\_129SRS098881\_contig\_number\_17771
817
species
5

1
species
1907658

SRS014948\_contig\_number\_13333

species
5
820

SRS017103\_contig\_number\_17698SRS021219\_contig\_number\_661SRS054905\_contig\_number\_94SRS056259\_contig\_number\_11415SRS147377\_contig\_number\_123

1946088

SRS147346\_contig\_number\_121
species
1


SRS144362\_contig\_number\_1102
329854
1
species


SRS063190\_contig\_number\_314
469590
species
1

1
species
469585

SRS018623\_contig\_number\_565

821

SRS011239\_contig\_number\_692SRS011405\_contig\_number\_533SRS012969\_contig\_number\_5427SRS013158\_contig\_number\_841SRS013382\_contig\_number\_26SRS013521\_contig\_number\_60SRS013687\_contig\_number\_1471SRS013965\_contig\_number\_306SRS014287\_contig\_number\_2297SRS014313\_contig\_number\_1057SRS014534\_contig\_number\_272SRS014855\_contig\_number\_441SRS014979\_contig\_number\_14062SRS015190\_contig\_number\_1991SRS015217\_contig\_number\_178SRS015369\_contig\_number\_8850SRS015431\_contig\_number\_35506SRS015486\_contig\_number\_358SRS015694\_contig\_number\_19940SRS015794\_contig\_number\_13001SRS015854\_contig\_number\_2046SRS016056\_contig\_number\_596SRS016095\_contig\_number\_contig-100\_869.156927SRS016132\_contig\_number\_776SRS016203\_contig\_number\_8SRS016495\_contig\_number\_662SRS016753\_contig\_number\_200SRS016840\_contig\_number\_1335SRS016897\_contig\_number\_139SRS016954\_contig\_number\_24732SRS016989\_contig\_number\_14711SRS017307\_contig\_number\_75SRS017375\_contig\_number\_469SRS017521\_contig\_number\_784SRS017745\_contig\_number\_743SRS018351\_contig\_number\_353SRS018836\_contig\_number\_20714SRS018888\_contig\_number\_1640SRS018936\_contig\_number\_144SRS019068\_contig\_number\_96709SRS019161\_contig\_number\_13628SRS019178\_contig\_number\_858SRS019381\_contig\_number\_14048SRS019808\_contig\_number\_12618SRS019968\_contig\_number\_137SRS020508\_contig\_number\_20SRS021484\_contig\_number\_918SRS021948\_contig\_number\_27896SRS022713\_contig\_number\_1195SRS023526\_contig\_number\_9720SRS023914\_contig\_number\_2558SRS023971\_contig\_number\_143SRS024009\_contig\_number\_183SRS024492\_contig\_number\_10618SRS024625\_contig\_number\_4016SRS042284\_contig\_number\_135SRS042966\_contig\_number\_15596SRS043411\_contig\_number\_65SRS043667\_contig\_number\_516SRS044535\_contig\_number\_1176SRS045004\_contig\_number\_11913SRS045244\_contig\_number\_809SRS045645\_contig\_number\_144SRS045739\_contig\_number\_269SRS045826\_contig\_number\_12561SRS046068\_contig\_number\_574SRS046369\_contig\_number\_12546SRS046502\_contig\_number\_13709SRS046810\_contig\_number\_contig-100\_12.13SRS047014\_contig\_number\_21597SRS047044\_contig\_number\_977SRS048164\_contig\_number\_81SRS048164\_contig\_number\_23200SRS049402\_contig\_number\_63SRS049446\_contig\_number\_693SRS049712\_contig\_number\_9554SRS049896\_contig\_number\_11863SRS049959\_contig\_number\_82SRS049995\_contig\_number\_28050SRS050299\_contig\_number\_1258SRS050422\_contig\_number\_9696SRS050520\_contig\_number\_1206SRS050752\_contig\_number\_35SRS050801\_contig\_number\_contig-100\_282.28329SRS050974\_contig\_number\_contig-100\_465.24504SRS050998\_contig\_number\_7090SRS051031\_contig\_number\_22669SRS051610\_contig\_number\_186SRS052027\_contig\_number\_14442SRS052576\_contig\_number\_191SRS052697\_contig\_number\_contig-100\_566.255103SRS053087\_contig\_number\_2613SRS053214\_contig\_number\_62SRS053335\_contig\_number\_158SRS053356\_contig\_number\_contig-100\_637.268152SRS053398\_contig\_number\_3381SRS054352\_contig\_number\_2647SRS056273\_contig\_number\_511SRS056537\_contig\_number\_53884SRS057478\_contig\_number\_6248SRS057480\_contig\_number\_8307SRS058268\_contig\_number\_6SRS058723\_contig\_number\_2910SRS062427\_contig\_number\_193SRS062654\_contig\_number\_284SRS062701\_contig\_number\_4331SRS063370\_contig\_number\_10548SRS063813\_contig\_number\_5612SRS063928\_contig\_number\_741SRS064973\_contig\_number\_168SRS065176\_contig\_number\_12737SRS065504\_contig\_number\_39SRS075078\_contig\_number\_2439SRS075716\_contig\_number\_330SRS075773\_contig\_number\_8930SRS075821\_contig\_number\_299SRS075984\_contig\_number\_21290SRS076756\_contig\_number\_16451SRS076876\_contig\_number\_123SRS077194\_contig\_number\_26593SRS077231\_contig\_number\_149SRS077294\_contig\_number\_958SRS077335\_contig\_number\_107SRS077392\_contig\_number\_33058SRS077589\_contig\_number\_4298SRS077730\_contig\_number\_714SRS077849\_contig\_number\_10482SRS077991\_contig\_number\_contig-100\_3.23910SRS078176\_contig\_number\_14426SRS078665\_contig\_number\_4305SRS097889\_contig\_number\_13781SRS097920\_contig\_number\_30462SRS097958\_contig\_number\_47SRS098061\_contig\_number\_113SRS098073\_contig\_number\_3265SRS098571\_contig\_number\_2789SRS098644\_contig\_number\_122SRS098827\_contig\_number\_215SRS100021\_contig\_number\_25SRS100907\_contig\_number\_contig-100\_97.62539SRS101376\_contig\_number\_5891SRS103987\_contig\_number\_21122SRS1041036\_contig\_number\_1050SRS1041039\_contig\_number\_8909SRS1041090\_contig\_number\_contig-100\_442.54285SRS1041092\_contig\_number\_133SRS1041095\_contig\_number\_contig-100\_1528.35625SRS1041116\_contig\_number\_3959SRS1041129\_contig\_number\_98SRS1041130\_contig\_number\_357SRS1041133\_contig\_number\_21335SRS1041134\_contig\_number\_245SRS1041137\_contig\_number\_1523SRS1041138\_contig\_number\_1321SRS1041139\_contig\_number\_3405SRS1041140\_contig\_number\_19819SRS1041142\_contig\_number\_3816SRS1041143\_contig\_number\_1970SRS1041144\_contig\_number\_4243SRS1041147\_contig\_number\_3097SRS104327\_contig\_number\_630SRS104485\_contig\_number\_400SRS104912\_contig\_number\_contig-100\_512.120435SRS104975\_contig\_number\_65SRS105082\_contig\_number\_160SRS1054716\_contig\_number\_292SRS1054928\_contig\_number\_3489SRS1055022\_contig\_number\_1007SRS1055049\_contig\_number\_234SRS1055056\_contig\_number\_contig-100\_295.77495SRS1055069\_contig\_number\_1870SRS1055076\_contig\_number\_1915SRS140492\_contig\_number\_41SRS140513\_contig\_number\_1487SRS142503\_contig\_number\_17006SRS142542\_contig\_number\_121SRS142781\_contig\_number\_126SRS142923\_contig\_number\_8642SRS143085\_contig\_number\_1086SRS143417\_contig\_number\_15405SRS143722\_contig\_number\_11154SRS143780\_contig\_number\_203SRS143876\_contig\_number\_18582SRS143895\_contig\_number\_44414SRS143991\_contig\_number\_18066SRS144183\_contig\_number\_24108SRS144297\_contig\_number\_173SRS144506\_contig\_number\_35610SRS144537\_contig\_number\_31229SRS144603\_contig\_number\_1804SRS144714\_contig\_number\_12994SRS144753\_contig\_number\_11694SRS145308\_contig\_number\_446SRS145497\_contig\_number\_10241SRS146764\_contig\_number\_162SRS147022\_contig\_number\_563SRS147039\_contig\_number\_2502SRS147557\_contig\_number\_34292SRS147614\_contig\_number\_302SRS147653\_contig\_number\_2311SRS147977\_contig\_number\_119SRS148091\_contig\_number\_578SRS148196\_contig\_number\_41587SRS148253\_contig\_number\_98SRS148424\_contig\_number\_18751SRS148721\_contig\_number\_32382SRS148970\_contig\_number\_1772SRS149075\_contig\_number\_2735SRS149181\_contig\_number\_16147SRS149879\_contig\_number\_20521SRS892882\_contig\_number\_14844SRS893170\_contig\_number\_360SRS893187\_contig\_number\_17258SRS893252\_contig\_number\_13206SRS893253\_contig\_number\_1021SRS893256\_contig\_number\_570SRS893259\_contig\_number\_4755SRS893279\_contig\_number\_69SRS893285\_contig\_number\_302SRS893288\_contig\_number\_580SRS893295\_contig\_number\_691SRS893300\_contig\_number\_234SRS893342\_contig\_number\_1131SRS893358\_contig\_number\_9454SRS893366\_contig\_number\_7492SRS893369\_contig\_number\_9269SRS893373\_contig\_number\_11431SRS893378\_contig\_number\_19876SRS893380\_contig\_number\_413SRS893382\_contig\_number\_659SRS893383\_contig\_number\_35354SRS971276\_contig\_number\_4124
species
232

species
1

SRS018427\_contig\_number\_2922
310297

1262739

SRS148159\_contig\_number\_16154
1
species


SRS011134\_contig\_number\_15878
2292941
1
species


SRS143342\_contig\_number\_97SRS149325\_contig\_number\_2848SRS893270\_contig\_number\_23932
246787
species
3

5
species

SRS017247\_contig\_number\_247SRS074964\_contig\_number\_1929SRS077502\_contig\_number\_16197SRS1055095\_contig\_number\_242SRS148817\_contig\_number\_2740
28116

species
1
291644

SRS074670\_contig\_number\_87

species
1

SRS017859\_contig\_number\_170
93975

1
species

SRS018049\_contig\_number\_694
357276

3
species
46506

SRS077454\_contig\_number\_221SRS143070\_contig\_number\_15SRS147425\_contig\_number\_99

2005525
2
family

genus
2
375288

species
2

SRS048164\_contig\_number\_11116SRS064757\_contig\_number\_76
46503

family
1
1853231

283168
1
genus

28118

SRS077502\_contig\_number\_590
1
species

1
family
171552

838
1
genus

1262933

SRS015663\_contig\_number\_1290
1
species
